# Indirect assessment of hemorrhoid incidence using invasive treatment data in Japan: A 5‐year study based on nationwide health insurance claims

**DOI:** 10.1002/ags3.70018

**Published:** 2025-03-25

**Authors:** Masamitsu Kido, Tomohiro Arita, Katsutoshi Shoda, Ken Inoue, Hiroyuki Okimura, Hiroki Shimizu, Jun Kiuchi, Kenji Nanishi, Atsushi Shiozaki

**Affiliations:** ^1^ Department of Orthopedic Surgery Inage Hospital Chiba Japan; ^2^ Division of Digestive Surgery, Department of Surgery, Graduate School of Medical Science Kyoto Prefectural University of Medicine Kyoto Japan; ^3^ First Department of Surgery, Faculty of Medicine University of Yamanashi Yamanashi Japan; ^4^ Department of Molecular Gastroenterology and Hepatology, Graduate School of Medical Science Kyoto Prefectural University of Medicine Kyoto Japan; ^5^ Department of Obstetrics and Gynecology, Graduate School of Medical Science Kyoto Prefectural University of Medicine Kyoto Japan

**Keywords:** database, epidemiology, hemorrhoids, invasive treatment, Japan

## Abstract

**Aim:**

The epidemiology of hemorrhoids is challenging because of variability in sampling methodologies and diagnostic criteria across different studies. This study indirectly clarified the epidemiology of hemorrhoids by investigating the number of invasive treatments for hemorrhoids (ITH) using a nationwide healthcare claims database.

**Methods:**

The annual number and rate of ITH procedures per 100 000 people during 2018–2022 were examined. ITH covered by the Japanese healthcare insurance includes sclerotherapy, rubber band ligation, infrared coagulation, thrombectomy, hemorrhoidectomy, and stapled hemorrhoidopexy. The demographic peak patterns in the sex‐ and age‐stratified data were analyzed. Annual trends were evaluated using the Jonckheere–Terpstra trend test or Poisson regression model.

**Results:**

Over the 5‐year period, the total number of ITH was 697 838, with a rate of 110.5 per 100 000 person‐years. The male‐to‐female ratio was 1.2:1, indicating slight male predominance. Demographic peak analysis revealed bimodal peaks in males aged 40–44 and 75–79 years, and in females aged 35–39 and 75–79 years. No significant changes were observed in all ITH procedures. The age‐adjusted number across all age groups per 100 000 person‐years demonstrated annual decreasing trends for males but increasing trends for females (*p* < 0.0167). Subgroup analysis indicated a decrease among older cohorts in both males and females, in contrast to an increase among young and middle‐aged females (*p* < 0.00088).

**Conclusion:**

Distinct bimodal ITH peaks were observed in males and females in their 30s–40s and 70s. These findings provide valuable insights into the epidemiology of hemorrhoids.

## Graphical abstract

This study aimed to clarify the epidemiology of hemorrhoids by leveraging a comprehensive nationwide healthcare claims database in Japan. By analyzing invasive treatments for hemorrhoids over a 5‐year period (2018–2022), we identified key demographic patterns, including distinct bimodal peaks in incidence among males and females, and highlighted evolving trends in treatment utilization. These findings enhance our understanding of invasive treatments for hemorrhoids and may inform the clinical practice of gastrointestinal surgeons.

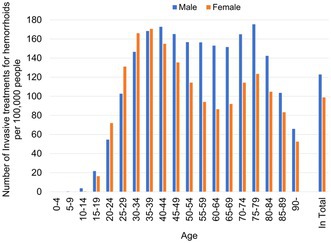

## 1 INTRODUCTION

Hemorrhoids are prevalent benign anorectal diseases.^1^, ^2^ The hemorrhoid‐related economic burden was estimated to be $800 million annually in the limited U.S. employer‐insured population aged 18–64 years.^3^ The epidemiology of hemorrhoids varies widely, as highlighted by a 2023 systematic review.^4^ In particular, the prevalence is reported to be 1.2% based on the above‐mentioned commercial claims database,^3^ 4.4%–14.4% from self‐reported patient surveys,^5^, ^6^, ^7^ 13.1% through physician examinations using anoscopy,^8^ and 16.6%–38.9% identified via screening colonoscopies.^9^, ^10^, ^11^ Furthermore, the incidence ranges from 708 to 1225 per 100 000 person‐years.^12^, ^13^ These disparities in epidemiological data stem primarily from variations in sampling methodologies and diagnostic criteria.

Given the pivotal role of gastrointestinal surgeons in the diagnosis and management of hemorrhoids, advancing epidemiological precision is crucial. Large‐scale databases present a potential solution for these sampling discrepancies. Japan's healthcare insurance system, which is characterized by universal health coverage,^14^ facilitates unrestricted access to medical services, thereby fostering the development of a comprehensive medical claims database. The National Database of Health Insurance Claims and Specific Health Checkups of Japan (NDB) encompasses more than 95% of the nation's medical insurance claims,^15^, ^16^, ^17^ rendering this real‐world dataset a robust resource for addressing sampling issues in epidemiological research.

The therapeutic options for hemorrhoids are diverse.^1^, ^2^, ^18^, ^19^, ^20^ When conservative treatments, such as lifestyle modifications and topical or oral medications, are ineffective, invasive treatments are recommended in accordance with local clinical practice guidelines.^18^, ^19^, ^20^ These invasive procedures, covered by the Japanese healthcare insurance, include sclerotherapy, rubber band ligation, infrared coagulation, thrombectomy, hemorrhoidectomy, and stapled hemorrhoidopexy.^18^ Each treatment modality has inherent limitations, including relatively high recurrence rates and potential complications, and no single approach has been universally accepted as the gold standard. Despite the availability of global treatment guidelines,^18^, ^19^, ^20^ geographical variations in therapeutic practices persist. However, nationwide observational epidemiological studies on the invasive treatments for hemorrhoids (ITH) are scarce.

This study aimed to elucidate the recent trends in the utilization of ITH in Japan using the NDB. Although this investigation focused on cases with severe symptoms rather than the general prevalence of the disease, the definitive diagnosis of ITH was based on macroscopic findings. Consequently, this approach may overcome the aforementioned challenges related to sampling and diagnosis in hemorrhoid epidemiology. Additionally, this study aimed to delineate demographic patterns associated with ITH, thereby providing critical insights into hemorrhoidal management trends and informing the clinical practice of gastrointestinal surgeons.

## 2 METHODS

### 2.1 Study design and data sampling technique

According to the Japanese ethical guidelines, this study did not require Institutional Review Board approval or informed consent because all data were obtained from publicly available sources. To ensure patient anonymity, the NDB Open Data Japan does not report data for procedures that occur fewer than 10 times per year.^21^

Utilizing the publicly available database of the NDB Open Data Japan,^21^ the sex‐ and age‐stratified (in 5‐year increments) numbers of ITH from 2018 to 2022 were annually analyzed. Data from 2014 to 2017 were excluded because of frequent changes in the definition of ITH procedures covered by the Japanese healthcare insurance. Demographic data from the Ministry of Internal Affairs and Communications^22^ were employed to compute the ITH rate per 100 000 people. ITH was further categorized according to the management type (outpatient, inpatient, or all). ITH was included using the following procedure codes:

K743 ITH.

1. Sclerotherapy (one‐shot).
2. Sclerotherapy (four‐step injection method).
3. Thrombectomy, (3‐1) Rubber band ligation, (3‐2) Infrared coagulation.
4. Hemorrhoidectomy without sclerotherapy (four‐step injection method).
5. Hemorrhoidectomy with sclerotherapy (four‐step injection method).
6. Stapled hemorrhoidopexy.

A400 short stay surgery for ITH.

1. Sclerotherapy (four‐step injection method).

The total number of sclerotherapies was defined as the sum of both one‐shot and four‐step injection methods [categorized as sclerotherapy (total), with codes K743 (1), (2), and A400 (1)]. The total number of hemorrhoidectomies included both with and without sclerotherapy (four‐step injection method) [categorized as hemorrhoidectomy (total), with codes K743 (4), (5)].

In Japan, sclerotherapy is administered using either a one‐shot or four‐step injection method.^18^, ^23^ Typically, 5% phenol in almond oil solution is used for one‐shot sclerotherapy, whereas aluminum potassium sulfate hydrate and tannic acid are used for the four‐step injection method.^23^ Since 2018, hemorrhoidectomy combined with sclerotherapy has been covered by the Japanese healthcare insurance, as it is considered to reduce recurrence rates.^24^

### 2.2 Data analyses

The overall male‐to‐female ratio across all procedures and age groups was calculated over the 5‐year period. A detailed breakdown of the mode share of the ITH procedures was provided using the most recent dataset from 2022. The proportion of each procedure type across the 10‐year age groups was also analyzed for the same year.

Demographic peak analysis of sex‐ and age‐stratified ITH rates (all procedures) per 100 000 people was conducted over the 5‐year study period.

Annual trends were evaluated for both the number and rate of ITH procedures from 2018 to 2022. Annual trends in the number of ITH, categorized by management type and procedure, were analyzed using the Jonckheere–Terpstra trend test. Additionally, annual trends in the number rate of ITH were performed using fitting Poisson regression models. The risk ratio (RR) for ITH was evaluated across all age groups and within age‐stratified subgroups. The following conditions were evaluated: all age groups and age‐stratified subgroups for both sexes, males, and females.

### 2.3 Statistical analyses

The data were evaluated after adjusting for age by the direct method using the standard age structure derived from the 2015 Population Census in Japan.^17^ The Jonckheere–Terpstra trend test was used to determine whether the number of ITH increased or decreased over the 5‐year study period.

Poisson regression models were constructed to examine the annual trends in the age‐adjusted ITH rate per 100 000 people. The number of ITH was set as the objective variable and the observation time point (year) and sex were set as the explanatory variables. Additionally, the population at each time point was considered by adding the person‐year population to the model as an offset. The number was also adjusted for age by the direct method, using the same age structure.^17^ Moreover, annual trend analysis of the subgroups was performed using age‐ and sex‐stratified samples.

All statistical analyses were performed using R (version 3.6.2, The R Foundation for Statistical Computing, Vienna, Austria). Statistical significance was set at two‐sided *p* values <0.05 for the Jonckheere–Terpstra trend test, and <0.0167 (0.05/3) and <0.00088 (0.05/57) for Poisson regression models for all‐age and age‐stratified analyses, respectively, using the Bonferroni's correction method for annual trend analysis.

## 3 RESULTS

### 3.1 Comprehensive analysis of ITH

The cumulative crude number of ITH across all procedures and age groups was 697 838 between 2018 and 2022. The annual average number of ITH procedures was 110.5 per 100 000 person‐years. For the breakdown, the annual average numbers were 28.2 for sclerotherapy (total) and 53.9 for hemorrhoidectomy (total) per 100 000 person‐years. The overall male‐to‐female ratio was 1.2:1, indicating slight male predominance.

### 3.2 Mode share and proportion of each ITH procedure in 2022

**FIGURE 1:**
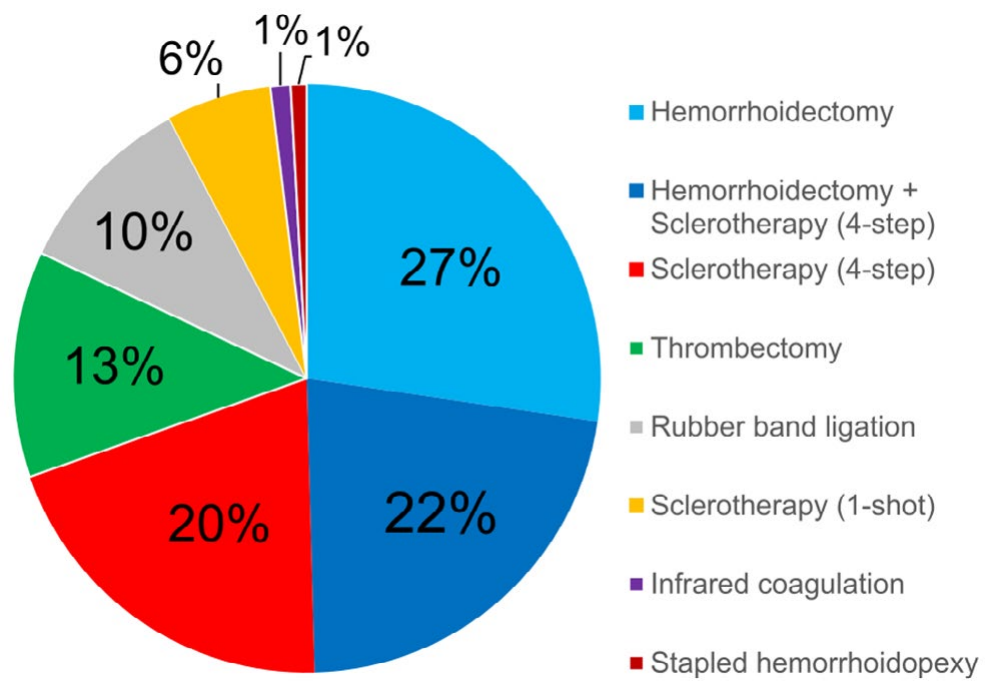
Annual mode share of invasive treatments for hemorrhoids in 2022. Hemorrhoidectomy (total) included both hemorrhoidectomy with and without sclerotherapy (four‐step injection method) [codes K743 (4), (5)]. Sclerotherapy (total) included both sclerotherapy (one‐shot) and sclerotherapy (four‐step injection method) [codes K743 (1), (2) and A400 (1)].

In 2022, the latest mode share of ITH procedures was as follows: sclerotherapy (one‐shot) (5.8%, 7897/137 136), sclerotherapy (four‐step) (20.0%, 27 406/137 136), rubber band ligation (10.2%, 14 045/137 136), infrared coagulation (1.1%, 1521/137 136), thrombectomy (12.5%, 17 080/137 136), hemorrhoidectomy without sclerotherapy (27.3%, 37 488/137 136), hemorrhoidectomy with sclerotherapy (22.2%, 30 498/137 136), and stapled hemorrhoidopexy (0.9%, 1201/137 136) (Figure 1).

**FIGURE 2:**
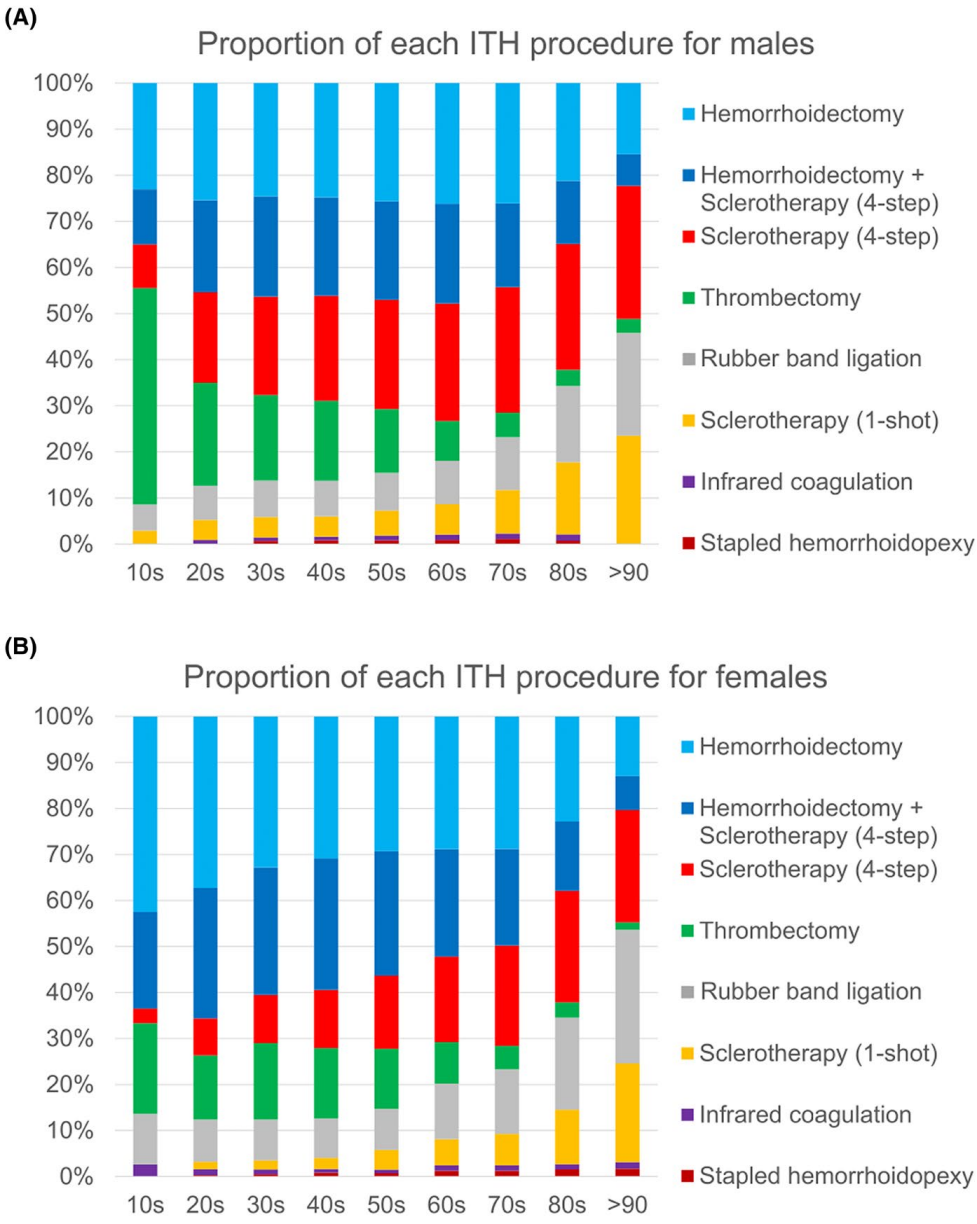
Proportion of each type of procedure for invasive treatments for hemorrhoids (ITH) in the 10‐year age groups for males (A) and females (B) in 2022.

Additionally, the proportion of each procedure type across the 10‐year age groups for males and females in 2022 is illustrated in Figure 2, offering a detailed demographic breakdown of procedure utilization patterns. Hemorrhoidectomy is the most commonly performed procedure in males (Figure 2A), particularly among young and middle‐aged patients (20s–60s). Sclerotherapy is also frequently used, increasing prevalence with age. Thrombectomy is predominantly performed in younger individuals, with the highest proportion observed in those in their 10s. Rubber band ligation is less frequently used but shows a slight increase with age. Infrared coagulation and stapled hemorrhoidopexy are the least commonly performed procedures.

In females (Figure 2B), the distribution of procedures is similar to that observed in males, with some variations. Hemorrhoidectomy remains the most frequently performed treatment, especially among younger patients. Sclerotherapy is also widely used, increasing in prevalence with age. Thrombectomy is commonly performed in younger individuals. Rubber band ligation is consistently utilized across all age groups, with a notable increase among patients aged 80 and older. Infrared coagulation and stapled hemorrhoidopexy remain the least frequently performed procedures.

Overall, the data revealed an age‐dependent transition in procedure selection: younger patients typically underwent more invasive procedures, such as hemorrhoidectomy, while less invasive options, such as sclerotherapy and rubber band ligation, were more frequently chosen for older patients.

### 3.3 Demographic peak analysis of all ITH during 2018–2022

Throughout the study period, the age‐stratified number of ITH (all procedures) exhibited bimodal peak patterns in males and females. Bimodal peaks were observed in males aged 40–44 and 75–79 years, and in females aged 35–39 and 75–79 years (Figure 3). The trough between these peaks was more gradual in males than in females. The detailed breakdown data for each year are presented in Figures S1–S9.

**FIGURE 3:**
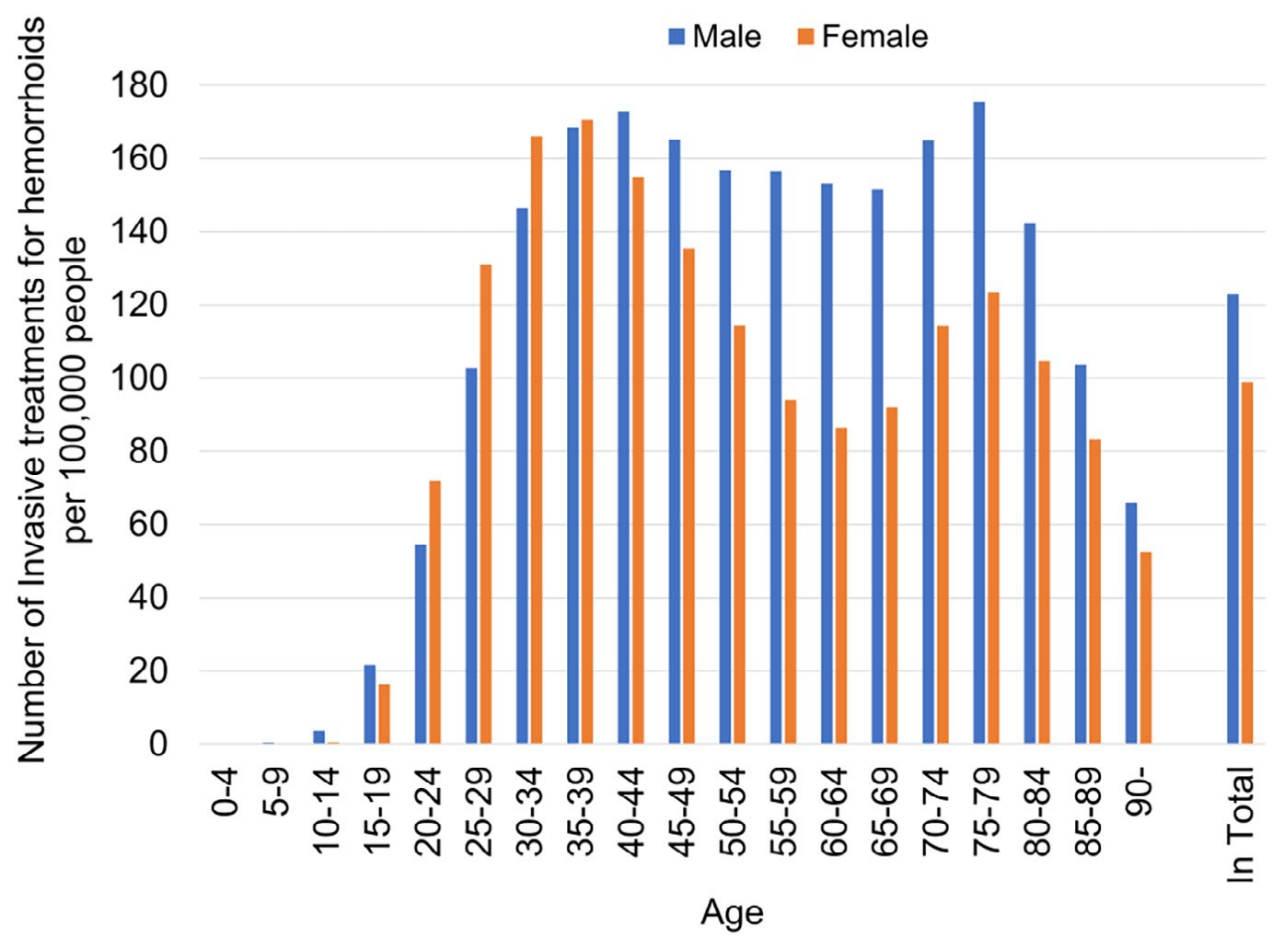
Age‐stratified number (5 years average) of invasive treatments for hemorrhoids (all procedures) per 100 000 people in Japan during 2018–2022.

### 3.4 Trends in the annual number of ITH from 2018 to 2022

**FIGURE 4:**
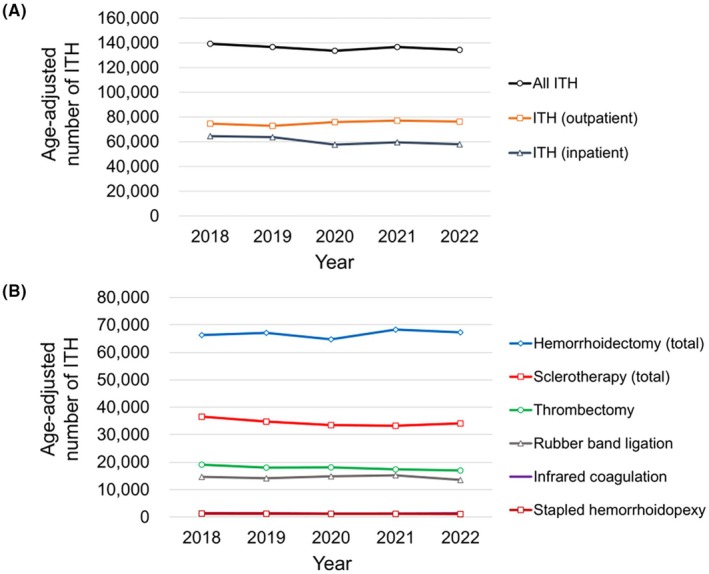
Trends in the annual age‐adjusted number of invasive treatments for hemorrhoids (ITH) in Japan from 2018 to 2022. (A) Trends in the annual number of ITH by management type (upper). (B) Trends in the annual number of ITH by procedure (lower). Hemorrhoidectomy (total) included both hemorrhoidectomy with and without sclerotherapy (four‐step injection method) [codes K743 (4), (5)]. Sclerotherapy (total) included both sclerotherapy (one‐shot) and sclerotherapy (four‐step injection method) [codes K743 (1), (2) and A400 (1)].

Over the 5‐year study period, no significant trends were observed in the age‐adjusted number of ITH for outpatients, inpatients, and all management types (*p* = 0.233, 0.233, and 0.233, respectively) (Figure 4A). Similarly, there were no significant changes in the number of specific ITH procedures, including sclerotherapy (total), rubber band ligation, infrared coagulation, thrombectomy, hemorrhoidectomy (total), and stapled hemorrhoidopexy (*p* = 0.233, 1, 0.483, 0.083, 0.483 and 0.083, respectively) (Figure 4B).

### 3.5 Trends in the annual number rate of ITH per 100 000 person‐years from 2018 to 2022

**FIGURE 5:**
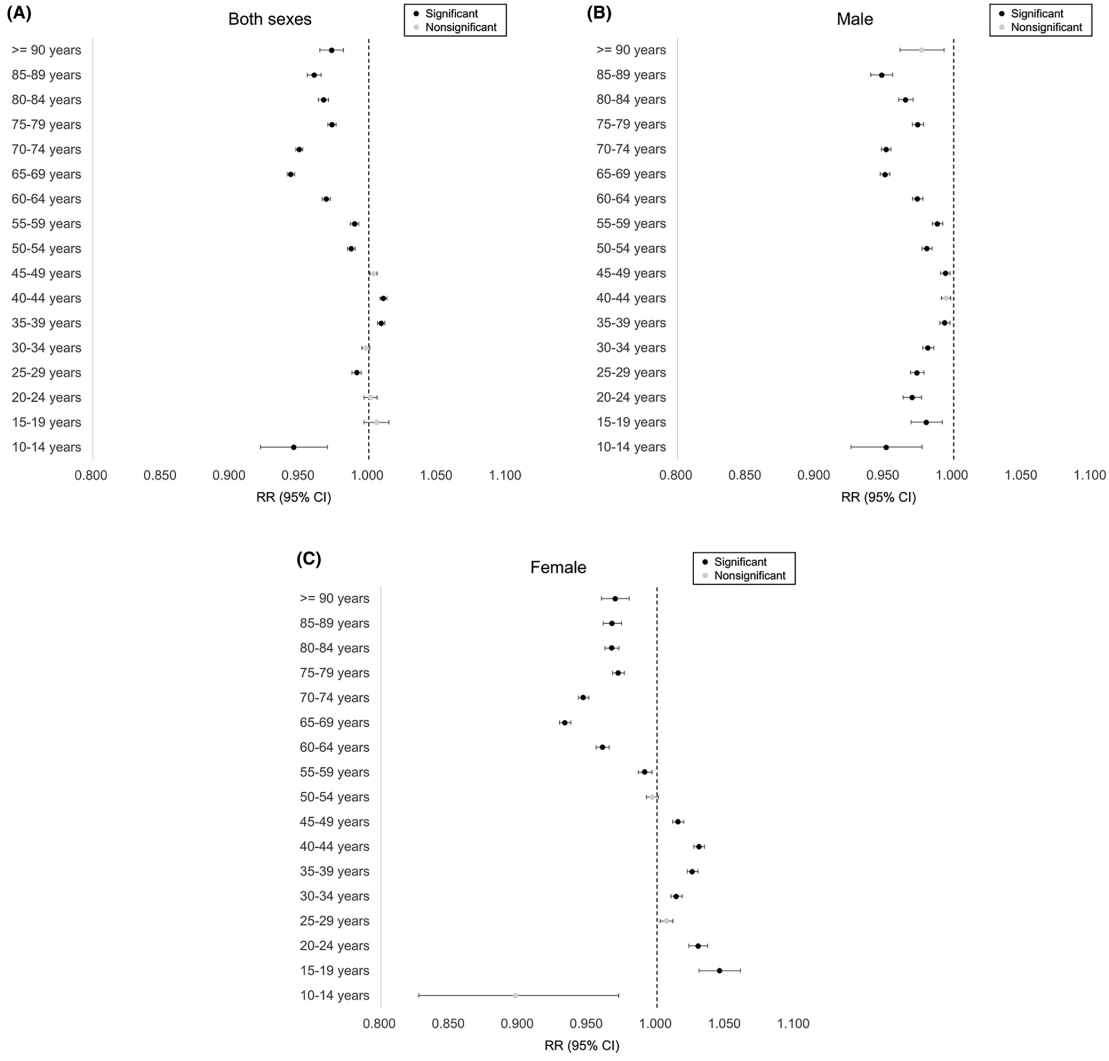
Risk ratio of the age‐stratified number of invasive treatments for hemorrhoids in both sexes (A), males (B), and females (C) per 100 000 person‐years using fitting Poisson regression models. Black dots indicate statistical significance, and gray dots indicate non‐significance. CI, confidence interval; RR, risk ratio.

The age‐adjusted ITH number across all age groups per 100 000 person‐years showed a decreasing annual trend for males, an increasing annual trend for females, and eventually a decreasing trend for both sexes (RR = 0.993, 0.984, and 1.003 for both sexes, males, and females, respectively; *p* < 0.0167) (Table 1). Subgroup analyses indicated decreasing trends in males across almost all ages and elderly females, and increasing trends in young and middle‐aged females (*p* < 0.00088; Figure 5 and Table 2).

**TABLE 1:** Annual trend analysis of the age‐adjusted number of invasive treatments for hemorrhoids with all ages per 100 000 person‐years by fitting Poisson regression models. (A) (Upper) Poisson regression model for both sexes, considering the effect of sex. (B) (Middle): Poisson regression model for males. (C) (Lower): Poisson regression model for females.

Variables	Levels	RR	95% CI	*p* Value
(A)				
Constant	–	1.201	1.196–1.206	<0.0001
Time point	–	0.993	0.991–0.995	<0.0001
(B)				
Constant	–	1.365	1.358–1.372	<0.0001
Time point	–	0.984	0.982–0.986	<0.0001
(C)				
Constant	–	1.052	1.046–1.059	< 0.0001
Time point	–	1.003	1.001–1.006	< 0.0167

Abbreviations: CI, confidence interval; RR, risk ratio.

**TABLE 2:** Annual trend analysis of the age‐stratified number of invasive treatments for hemorrhoids of both sexes, males and females per 100 000 person‐years by fitting Poisson regression models.

Age group	Sex	Constant			Time point		
		RR	95% CI	*p* Value	RR	95% CI	*p* Value
10–14	Both	0.031	0.028–0.034	<0.0001	0.946	0.921–0.970	<0.0001
10–14	Male	0.053	0.047–0.059	<0.0001	0.951	0.926–0.977	0.00029
10–14	Female	0.008	0.006–0.010	<0.0001	0.898	0.827–0.972	0.00859
15–19	Both	0.190	0.183–0.197	<0.0001	1.006	0.997–1.015	0.22656
15–19	Male	0.244	0.233–0.256	<0.0001	0.980	0.969–0.992	0.00083
15–19	Female	0.134	0.126–0.142	<0.0001	1.045	1.031–1.061	<0.0001
20–24	Both	0.640	0.627–0.653	<0.0001	1.001	0.996–1.006	0.60854
20–24	Male	0.656	0.638–0.675	<0.0001	0.970	0.963–0.977	<0.0001
20–24	Female	0.628	0.610–0.646	<0.0001	1.030	1.023–1.037	<0.0001
25–29	Both	1.248	1.230–1.266	<0.0001	0.991	0.988–0.995	<0.0001
25–29	Male	1.204	1.180–1.229	<0.0001	0.974	0.969–0.979	<0.0001
25–29	Female	1.296	1.270–1.321	<0.0001	1.007	1.002–1.012	0.00401
30–34	Both	1.583	1.565–1.602	<0.0001	0.998	0.995–1.001	0.16448
30–34	Male	1.622	1.595–1.649	<0.0001	0.982	0.978–0.986	<0.0001
30–34	Female	1.546	1.520–1.572	<0.0001	1.014	1.010–1.018	<0.0001
35–39	Both	1.608	1.590–1.625	<0.0001	1.009	1.006–1.012	<0.0001
35–39	Male	1.741	1.715–1.766	<0.0001	0.994	0.990–0.997	0.00084
35–39	Female	1.474	1.451–1.498	<0.0001	1.026	1.022–1.030	<0.0001
40–44	Both	1.532	1.516–1.548	<0.0001	1.011	1.008–1.013	<0.0001
40–44	Male	1.757	1.733–1.781	<0.0001	0.995	0.991–0.998	0.00158
40–44	Female	1.305	1.285–1.326	<0.0001	1.031	1.027–1.034	<0.0001
45–49	Both	1.442	1.426–1.458	<0.0001	1.003	1.001–1.006	0.01048
45–49	Male	1.666	1.641–1.690	<0.0001	0.994	0.991–0.998	0.00080
45–49	Female	1.217	1.196–1.237	<0.0001	1.015	1.011–1.019	<0.0001
50–54	Both	1.426	1.409–1.443	<0.0001	0.987	0.985–0.990	<0.0001
50–54	Male	1.707	1.681–1.733	<0.0001	0.981	0.977–0.984	<0.0001
50–54	Female	1.143	1.122–1.165	<0.0001	0.997	0.992–1.001	0.13826
55–59	Both	1.299	1.282–1.316	<0.0001	0.990	0.987–0.993	<0.0001
55–59	Male	1.639	1.613–1.666	<0.0001	0.988	0.985–0.992	<0.0001
55–59	Female	0.962	0.942–0.983	0.00034	0.991	0.986–0.996	0.00075
60–64	Both	1.376	1.360–1.393	<0.0001	0.969	0.966–0.972	<0.0001
60–64	Male	1.717	1.691–1.743	<0.0001	0.974	0.970–0.978	<0.0001
60–64	Female	1.048	1.028–1.068	<0.0001	0.961	0.956–0.965	<0.0001
65–69	Both	1.610	1.593–1.627	<0.0001	0.944	0.941–0.946	<0.0001
65–69	Male	1.923	1.896–1.950	<0.0001	0.950	0.947–0.954	<0.0001
65–69	Female	1.319	1.297–1.341	<0.0001	0.933	0.929–0.938	<0.0001
70–74	Both	1.794	1.775–1.814	<0.0001	0.950	0.947–0.952	<0.0001
70–74	Male	2.113	2.082–2.144	<0.0001	0.951	0.948–0.955	<0.0001
70–74	Female	1.518	1.493–1.543	<0.0001	0.947	0.943–0.951	<0.0001
75–79	Both	1.623	1.602–1.643	<0.0001	0.973	0.970–0.976	<0.0001
75–79	Male	1.921	1.887–1.954	<0.0001	0.974	0.970–0.978	<0.0001
75–79	Female	1.385	1.360–1.411	<0.0001	0.972	0.968–0.976	<0.0001
80–84	Both	1.383	1.362–1.405	<0.0001	0.967	0.963–0.971	<0.0001
80–84	Male	1.646	1.609–1.683	<0.0001	0.965	0.960–0.971	<0.0001
80–84	Female	1.208	1.183–1.235	<0.0001	0.967	0.962–0.972	<0.0001
85–89	Both	1.092	1.068–1.116	<0.0001	0.960	0.955–0.966	<0.0001
85–89	Male	1.324	1.279–1.370	<0.0001	0.948	0.940–0.956	<0.0001
85–89	Female	0.974	0.947–1.002	0.07463	0.968	0.961–0.974	<0.0001
≥90	Both	0.632	0.608–0.656	<0.0001	0.973	0.965–0.982	<0.0001
≥90	Male	0.746	0.694–0.802	<0.0001	0.977	0.961–0.993	0.00536
≥90	Female	0.598	0.572–0.625	<0.0001	0.970	0.960–0.980	<0.0001

Abbreviations: CI, confidence interval; RR, risk ratio.

## 4 DISCUSSION

Using a comprehensive national medical insurance claims database in Japan, an observational epidemiological study was conducted to analyze the number of ITH according to sex and age. Previous studies have rarely reported the epidemiology of ITH in detail. A previous study utilizing insurance databases from the United States and England and Wales reported an average annual incidence of 1164–1225 per 100 000 population for hemorrhoids.^13^ This included 1177 physician visits and 48 hospital discharges in the United States and 1123 physician visits and 41 hospital discharges in England and Wales between 1983 and 1987. The same study further identified 49–60 hemorrhoidectomies per 100 000 U.S. population, with the majority occurring among individuals aged 45–64 years (95–123 hemorrhoidectomies) when stratified into four age categories: <15, 15–44, 45–64, and >65 years.^13^ Our findings for hemorrhoidectomies (total), with an average annual rate of 53.9 per 100 000 person‐years, are comparable to these historical data. Furthermore, by using Japan's universal health insurance database, we were able to provide more granular data on ITH, including not only hemorrhoidectomy but also other procedures, segmented into 5‐year age groups. This allowed a more detailed analysis of the demographic peak patterns of ITH (Figures S1–S9).

The most recent data, presented in Figure 1, highlight the predominance of hemorrhoidectomy procedures (with or without sclerotherapy) and sclerotherapy (one‐shot or four‐step injection method) as primary invasive treatment approaches for hemorrhoids. Furthermore, the age‐specific distribution of ITH procedures, shown in Figure 2, demonstrates a clear shift from more invasive to less invasive treatments with increasing patient age. In the teenage group (10s), there is a higher proportion of thrombectomy procedures, particularly among boys, suggesting a higher prevalence of external hemorrhoids. In the young to middle‐aged group (20s–60s), treatment strategies become more diverse, reflecting an individualized approach tailored to patient‐specific factors. In the older age group (70s and above), there is a marked preference for less invasive procedures, likely influenced by increased medical risks and recovery capacity considerations in older patients. Notably, among younger patients (under 60), males tend to prefer less invasive treatments compared to females. These findings suggest that ITH treatment choices are shaped by a complex interplay of factors, including age, gender, lifestyle, social circumstances, underlying medical risks, and anticipated treatment outcomes. Understanding these patterns can provide valuable insights for optimizing age‐ and condition‐specific treatment strategies in clinical practice.

Our comprehensive demographic analysis of all ITH revealed bimodal peak patterns in both males and females at the national level in Japan, with peaks observed in both the middle‐aged and elderly populations (Figure 3). Previous epidemiological studies on hemorrhoids have predominantly reported monomodal peaks in middle‐aged^7^, ^13^ or elderly populations,^5^ and in some cases, trimodal peaks in middle‐aged, elderly, and super‐elderly populations.^11^ Monomodal peaks have typically been observed in studies using large‐scale data, such as nationwide surveys. However, these studies relied on low‐resolution data with age increments of >10 years or by grouping all individuals aged ≥60 years together. In contrast, trimodal peaks were identified in studies using high‐resolution data (5‐year increments) but were based on smaller sample sizes (*n* = 976). Our study, using a nationwide high‐resolution database with 5‐year increments, provides a more robust representation. A recent study^25^ further supported our findings by suggesting nonlinear aging patterns in molecular markers, with two local peaks around the ages of 44 and 60 years, which could explain the identified bimodal peaks. Additionally, the trough between these bimodal peaks was less pronounced in males than in females. Regarding risk factors for hemorrhoids, a 2023 systematic review identified sedentary behavior, constipation, male sex, and advancing age as significant contributors.^4^ The observed gender disparity could be explained by the gender gap in labor force participation, where men's employment follows a continuous mountain‐shaped curve, whereas women's participation exhibits an M‐shaped curve, reflecting periods of employment interruption, particularly due to childbirth and caregiving responsibilities.^26^, ^27^ Longer labor force participation in men may be associated with prolonged sedentary behavior, which is a known risk factor for hemorrhoids. However, the second demographic peak (75–79 years) may be attributable to the higher prevalence of less invasive ITH procedures, such as sclerotherapy and rubber band ligation, among older age groups (Figure 2), as these procedures have been shown to have higher recurrence and reoperation rates compared to hemorrhoidectomy.^18^ Further in‐depth investigation is necessary.

The sex distribution of ITH also showed distinct patterns in each age group. Females were dominant in their 20s and 30s, whereas males predominated in the younger age groups (teenagers and younger) and those aged ≥40 years (Figure 3). Pregnancy increases the risk of hemorrhoids in women.^6^, ^7^, ^9^ Pregnancy‐related venous stasis in the perianal region caused by increased circulating blood volume and elevated intra‐abdominal pressure has been proposed as a major contributing factor.^28^, ^29^ Furthermore, progesterone produced during pregnancy relaxes smooth muscles in both the venous walls and intestines, exacerbating constipation and promoting the development of hemorrhoids.^28^ The dominance of females in their 20s and 30s may further support these hypotheses, as the birth rate in Japan peaks at ages 30–34 years (Figure S10). However, many other factors not considered in this study may also have influenced the sex distribution of ITH.

Regarding the trends in all ITH procedures, no significant changes were observed (Figure 4A,B). This indicates that invasive treatments for hemorrhoids in Japan remained consistent over the 5‐year study period. However, the notable decrease in inpatient ITH in 2020 likely reflects the disruption of healthcare services and reduced patient access to treatment caused by the COVID‐19 pandemic.

Our detailed analysis of annual trends in ITH per 100 000 people demonstrated a decrease among males, but an increase among females (Table 1). The recent downward trend of ITH among nearly all age males and elderly females (Figure 5 and Table 2) may be attributed to the impact of the COVID‐19 pandemic, particularly among older populations. Additionally, advances in surgical techniques, such as the combination of hemorrhoidectomy with four‐step injection sclerotherapy, may have reduced the need for reoperation. Although the use of 3% polidocanol for sclerotherapy has become widespread worldwide,^30^ further comparative studies are needed to assess the relative efficacies of different injection agents and procedures. In contrast, the upward trend observed among young and middle‐aged females (Figure 5C) may be related to the increasing number of women in sedentary occupations, as more women are participating in desk jobs within the Japanese workforce.^26^ However, there are conflicting reports in the literature; one study cited above^4^ suggests that sedentary behavior is a risk factor for hemorrhoids, whereas another suggests the opposite.^10^ Alternatively, recent changes in social environments may have made ITH more accessible to young women. In addition, the birth rate per 1000 females aged 20–39 years has shown a decreasing trend (Figure S10). Further longitudinal studies are required to confirm this hypothesis.

This study has several limitations. First, our analysis focused on the number of ITH procedures, rather than the incidence of hemorrhoids. The gap between ITH and hemorrhoids may have influenced our analysis in terms of ITH accessibility. In addition, the clinical severity or background of the cases, such as Goligher classification, were not captured. Second, because these data refer to invasive treatments, they may not reflect the epidemiology of less severe hemorrhoids (e.g., Goligher stage 1 or 2), which are typically treated with lifestyle modifications and topical or oral medications. Third, the 5‐year study period may be considered relatively short because the definition of the procedure code (K743) changed frequently prior to 2017. Finally, the generalizability of our results to other countries may be limited because of significant differences in medical and social systems or racial compositions.

In conclusion, a detailed observational epidemiological study of ITH utilization was conducted using the Japanese nationwide health insurance claims database. From 2018 to 2022, the average annual number of ITH procedures was 110.5 per 100 000 person‐years. Our analysis identified distinct bimodal peaks in the 30s–40s and 70s age groups for both males and females, indirectly suggesting age‐related patterns in the epidemiology of hemorrhoids. The study also highlighted annual decreasing trends in ITH among males and the elderly of both sexes in contrast to an increasing trend among females, particularly among young and middle‐aged cohorts. These findings not only provide a comprehensive overview of ITH utilization in Japan but also serve as a valuable reference for informed decision‐making in ITH management.

## AUTHOR CONTRIBUTIONS

**Masamitsu Kido:** Conceptualization; data curation; formal analysis; investigation; methodology; writing – original draft; writing – review and editing. **Tomohiro Arita:** Conceptualization; writing – review and editing. **Katsutoshi Shoda:** Methodology; writing – review and editing. **Ken Inoue:** Writing – review and editing. **Hiroyuki Okimura:** Writing – review and editing. **Hiroki Shimizu:** Writing – review and editing. **Jun Kiuchi:** Writing – review and editing. **Kenji Nanishi:** Writing – review and editing. **Atsushi Shiozaki:** Supervision.

## FUNDING INFORMATION

This study did not receive any specific grants from funding agencies in the public, commercial, or non‐profit sectors.

## CONFLICT OF INTEREST STATEMENT

The authors declare no conflicts of interest for this article.

## ETHICS STATEMENT

Approval of the research protocol by an Institutional Reviewer Board: This study did not require institutional board approval or informed consent because of its retrospective nature and use of legally anonymized public data.

Informed Consent: N/A.

Registry and the Registration No. of the study/trial: N/A.

Animal Studies: N/A.

## Supporting information


Figure S1.

## References

[1] Mott T , Latimer K , Edwards C . Hemorrhoids: diagnosis and treatment options. Am Fam Physician. 2018;97(3):172–179. 29431977

[2] Sandler RS , Peery AF . Rethinking what we know about hemorrhoids. Clin Gastroenterol Hepatol. 2019;17(1):8–15. 29601902 10.1016/j.cgh.2018.03.020 PMC7075634

[3] Yang JY , Peery AF , Lund JL , Pate V , Sandler RS . Burden and cost of outpatient hemorrhoids in the United States employer‐insured population, 2014. Am J Gastroenterol. 2019;114(5):798–803. 30741736 10.14309/ajg.0000000000000143 PMC6502684

[4] Lohsiriwat V , Sheikh P , Bandolon R , Ren DL , Roslani AC , Schaible K , et al. Recurrence rates and pharmacological treatment for hemorrhoidal disease: a systematic review. Adv Ther. 2023;40(1):117–132. 36331754 10.1007/s12325-022-02351-7 PMC9859842

[5] Johanson JF , Sonnenberg A . The prevalence of hemorrhoids and chronic constipation. Gastroenterology. 1990;98(2):380–386. 2295392 10.1016/0016-5085(90)90828-o

[6] Sheikh P , Régnier C , Goron F , Salmat G . The prevalence, characteristics and treatment of hemorrhoidal disease: results of an international web‐based survey. J Comp Eff Res. 2020;9(17):1219–1232. 33079605 10.2217/cer-2020-0159

[7] Lee JH , Kim HE , Kang JH , Shin JY , Song YM . Factors associated with hemorrhoids in Korean adults: Korean national health and nutrition examination survey. Korean J Fam Med. 2014;35(5):227–236. 25309703 10.4082/kjfm.2014.35.5.227 PMC4192796

[8] Kibret AA , Oumer M , Moges AM . Prevalence and associated factors of hemorrhoids among adult patients visiting the surgical outpatient department in the University of Gondar comprehensive specialized hospital, Northwest Ethiopia. PLoS One. 2021;16(4):e0249736. 33878128 10.1371/journal.pone.0249736 PMC8057569

[9] Hong YS , Jung KU , Rampal S , Zhao D , Guallar E , Ryu S , et al. Risk factors for hemorrhoidal disease among healthy young and middle‐aged Korean adults. Sci Rep. 2022;12(1):129. 34996957 10.1038/s41598-021-03838-z PMC8741788

[10] Peery AF , Sandler RS , Galanko JA , Bresalier RS , Figueiredo JC , Ahnen DJ , et al. Risk factors for hemorrhoids on screening colonoscopy. PLoS One. 2015;10(9):e0139100. 26406337 10.1371/journal.pone.0139100 PMC4583402

[11] Riss S , Weiser FA , Schwameis K , Riss T , Mittlböck M , Steiner G , et al. The prevalence of hemorrhoids in adults. Int J Colorectal Dis. 2012;27(2):215–220. 21932016 10.1007/s00384-011-1316-3

[12] Lin LH , Siu JJY , Liao PC . Association of chronic obstructive pulmonary disease and hemorrhoids: a nationwide cohort study. Med Baltim. 2017;96(10):e6281. 10.1097/MD.0000000000006281 PMC5348194 28272246

[13] Johanson JF , Sonnenberg A . Temporal changes in the occurrence of hemorrhoids in the United States and England. Dis Colon Rectum. 1991;34(7):585–593. 1820756 10.1007/BF02049899

[14] GBD . Universal health coverage collaborators. Measuring universal health coverage based on an index of effective coverage of health services in 204 countries and territories, 1990–2019: a systematic analysis for the global burden of disease study 2019. Lancet. 2020;2019(396):1250–1284. 10.1016/S0140-6736(20)30750-9 PMC7562819 32861314

[15] Kido M , Ikoma K , Kobayashi Y , Sotozono Y , Uehara R , Takahashi K . Trends and age‐ and sex‐stratified analysis of hallux valgus correction surgery from 2014 to 2019: a nationwide population‐based cohort study in Japan. Foot Ankle Surg. 2023;29(8):584–587. 37438238 10.1016/j.fas.2023.06.009

[16] Kido M , Shoda K , Yan L , Ikoma K , Ichikawa D . Inter‐ prefectural regional disparities in gastric cancer surgery: a Japanese nationwide population‐ based cohort study from 2014 to 2019. Ann Gastroenterol Surg. 2024;00:1–9. 10.1002/ags3.12813 PMC11533019 39502726

[17] Kido M , Okada S , Takashima N , Yan L , Uchibori A , Sensaki K , et al. Inter‐prefectural and urban‐rural regional disparities in lung cancer surgery: a Japanese nationwide population‐based cohort study from 2017 to 2019. Surg Today. 2024;54:1428–1436. 38739174 10.1007/s00595-024-02864-4

[18] Yamana T . Japanese practice guidelines for anal disorders I. Hemorrhoids. J Anus Rect Colon. 2017;1(3):89–99. 10.23922/jarc.2017-018 PMC6768674 31583307

[19] van Tol RR , Kleijnen J , Watson AJM , Jongen J , Altomare DF , Qvist N , et al. European society of ColoProctology: guideline for haemorrhoidal disease. Colorectal Dis. 2020;22(6):650–662. 32067353 10.1111/codi.14975

[20] Hawkins AT , Davis BR , Bhama AR . The American Society of Colon and Rectal Surgeons clinical practice guidelines for the management of hemorrhoids. Dis Colon Rectum. 2024;67(5):614–623. 38294832 10.1097/DCR.0000000000003276

[21] Ministry of Health, Labour and Welfare, Open Data NDB . Accessed 25 June 2024. Available from: https://www.mhlw.go.jp/stf/seisakunitsuite/bunya/0000177182.html.

[22] Portal site of official statistics of Japan (e‐stat). Accessed 25 June 2024. Available from: https://www.e‐stat.go.jp/en/stat‐search/.

[23] Hachiro Y , Kunimoto M , Abe T , Kitada M , Ebisawa Y . Aluminum potassium sulfate and tannic acid (ALTA) injection as the mainstay of treatment for internal hemorrhoids. Surg Today. 2011;41(6):806–809. 21626327 10.1007/s00595-010-4386-x

[24] Gallo G , Laforgia R , Goglia M , Lobascio P . Sclerotherapy with 3% polidocanol foam for the treatment of mucocutaneous bridges and/or residual piles after open excisional hemorrhoidectomy. Updates Surg. 2024;76(5):2087–2090. 38480640 10.1007/s13304-024-01798-3

[25] Shen X , Wang C , Zhou X . Nonlinear dynamics of multi‐omics profiles during human aging. Nat Aging. 2024;4:1619–16. doi: 10.1038/s43587-024-00692-2 39143318 PMC11564093

[26] Gender Equality Bureau, Cabinet Office, Government of Japan . Chapter 2. Gender equality in employment. White paper on gender equality. 2020. In: Summary. Page 15–16. Accessed 27 June 2024. Available from: https://www.gender.go.jp/english_contents/about_danjo/whitepaper/pdf/ewp2020.pdf.

[27] Gender Equality Bureau, Cabinet Office, Government of Japan . Change in attitudes toward work, Current status and challenges of housework, childcare, etc. and work styles. 2023 The white paper on gender equality‐Section 1; p. 23. Accessed 27 June 2024. Available from: https://www.gender.go.jp/about_danjo/whitepaper/r05/gaiyou/pdf/r05_gaiyou_en.pdf.

[28] Shin GH , Toto EL , Schey R . Pregnancy and postpartum bowel changes: constipation and fecal incontinence. Am J Gastroenterol. 2015;110(4):521–529. 25803402 10.1038/ajg.2015.76

[29] Sanghavi M , Rutherford JD . Cardiovascular physiology of pregnancy. Circulation. 2014;130(12):1003–1008. 25223771 10.1161/CIRCULATIONAHA.114.009029

[30] Gallo G , Picciariello A , Armellin C . Sclerotherapy for hemorrhoidal disease: systematic review and meta‐analysis. Tech Coloproctol. 2024;28(1):28. 38261136 10.1007/s10151-023-02908-w PMC10806988

