# Supplementary material for: Indirect assessment of hemorrhoid incidence using invasive treatment data in Japan: A 5‐year study based on nationwide health insurance claims
Source: Ann Gastroenterol Surg. 2025 Mar 25;9(5):987–96. doi: 10.1002/ags3.70018 (PMC12414590; doi:10.1002/ags3.70018)
Supplement: Supplementary file 1 — Figure S1. [file AGS3-9-987-s001.docx]

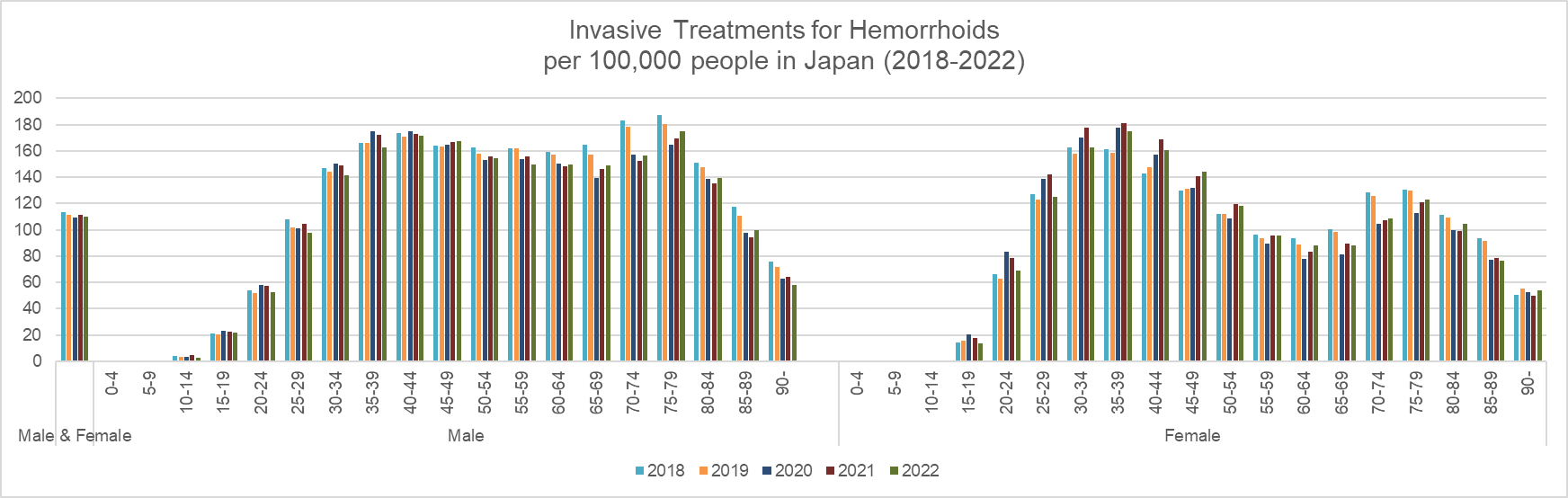


Supplementary Figure 1. Annual age-stratified number rate of invasive treatments for hemorrhoids (all procedures) per 100,000 people from 2018 to 2022 in Japan.


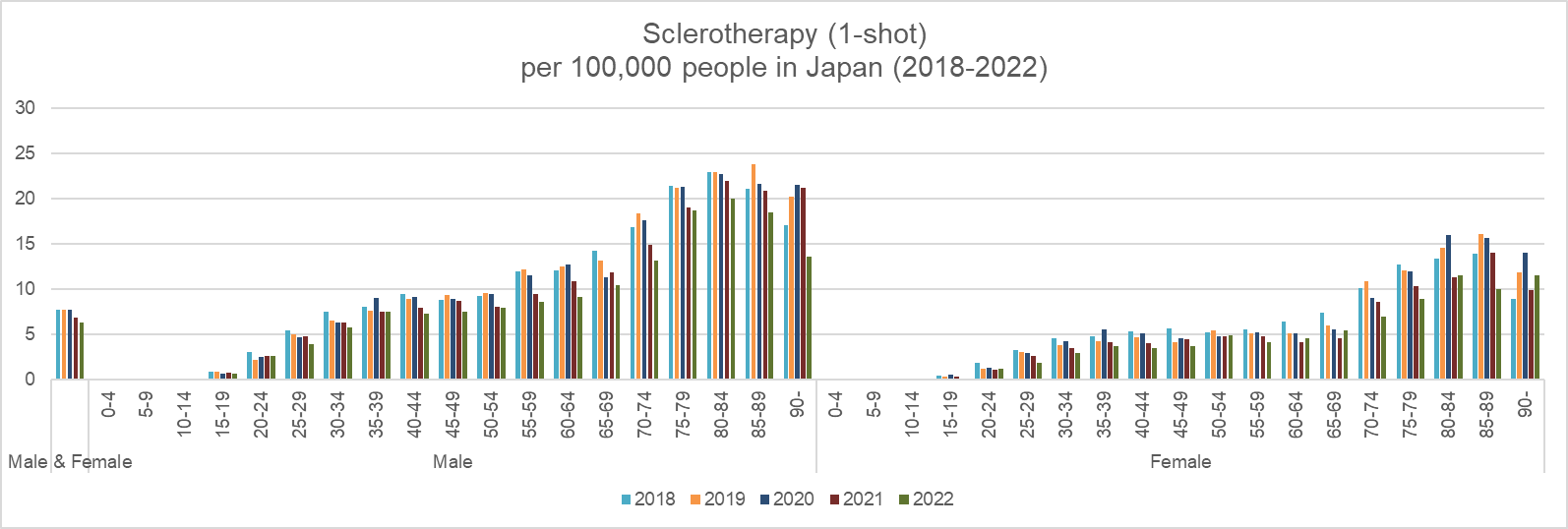


Supplementary Figure 2. Annual age-stratified number rate of invasive treatments for hemorrhoids (sclerotherapy (one-shot)) per 100,000 people from 2018 to 2022 in Japan.


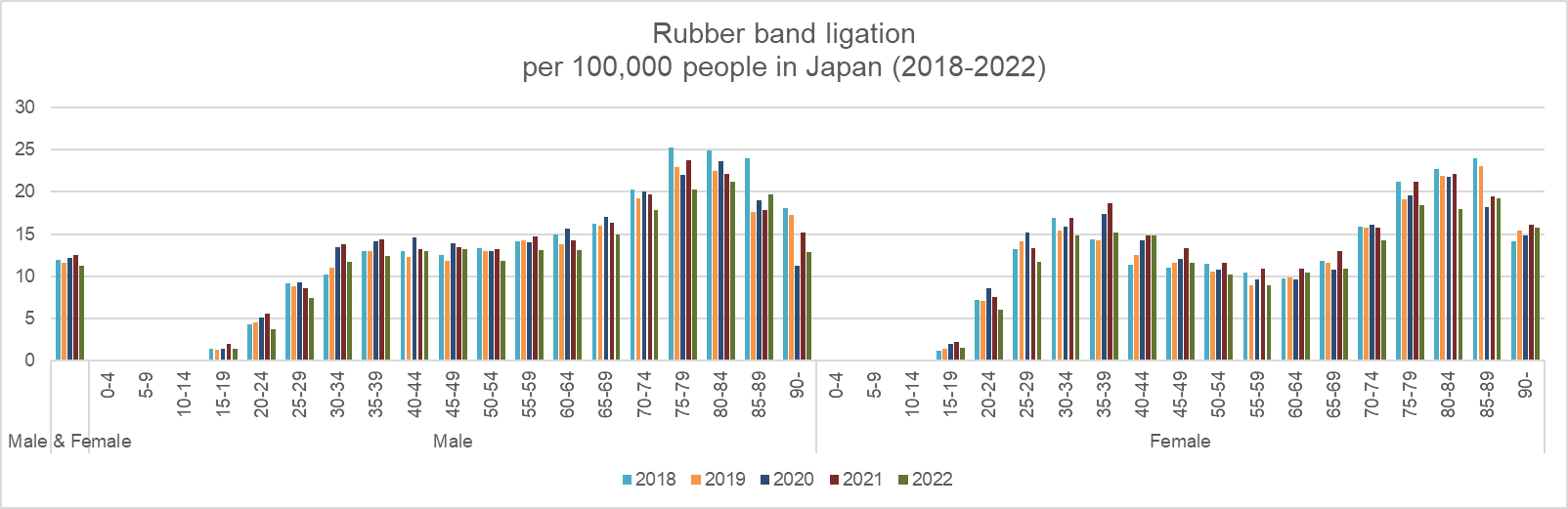


Supplementary Figure 3. Annual age-stratified number rate of invasive treatments for hemorrhoids (rubber band ligation) per 100,000 people from 2018 to 2022 in Japan.


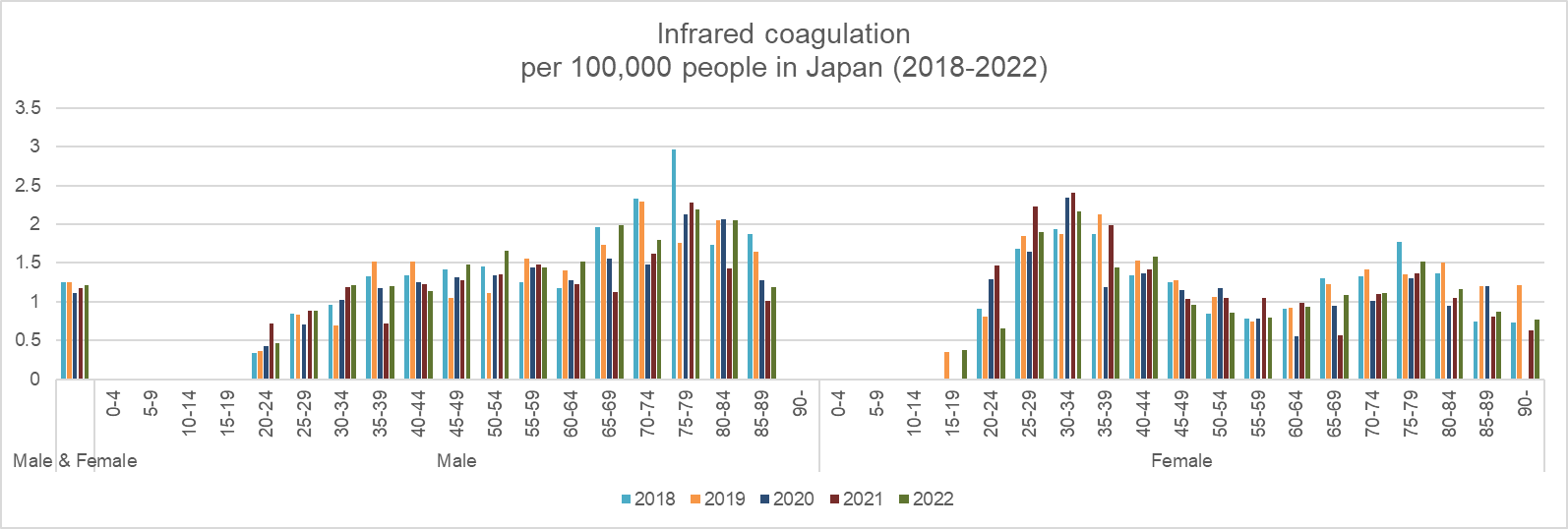


Supplementary Figure 4. Annual age-stratified number rate of invasive treatments for hemorrhoids (infrared coagulation) per 100,000 people from 2018 to 2022 in Japan.


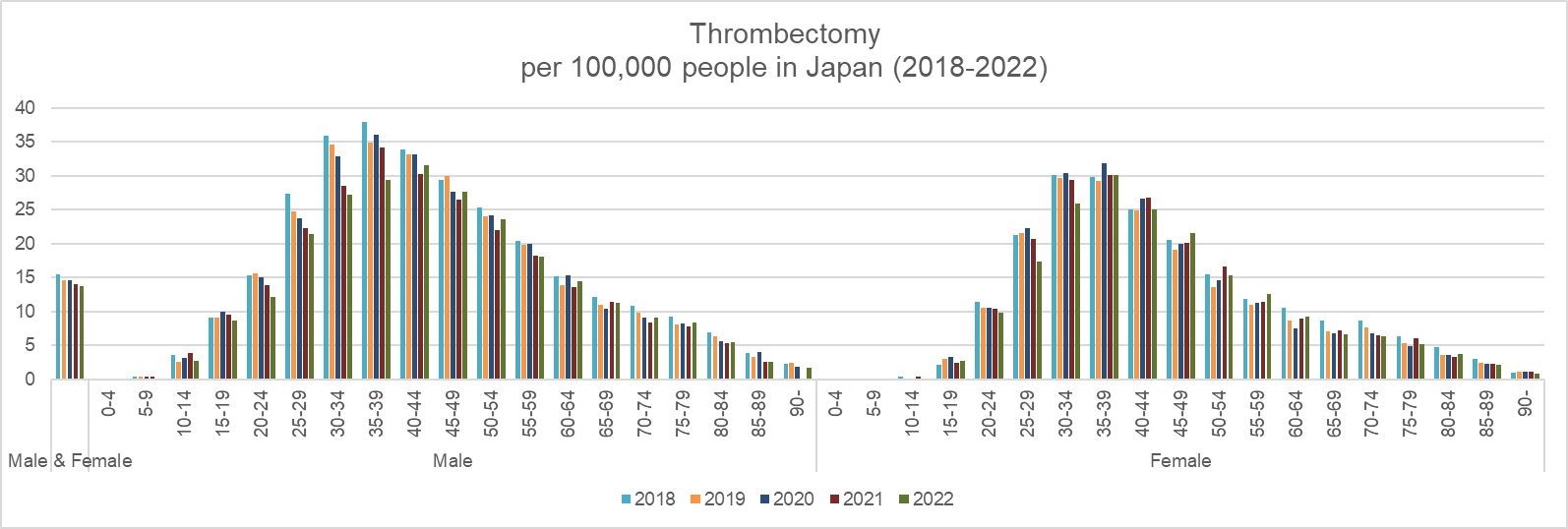


Supplementary Figure 5. Annual age-stratified number rate of invasive treatments for hemorrhoids (thrombectomy) per 100,000 people from 2018 to 2022 in Japan.


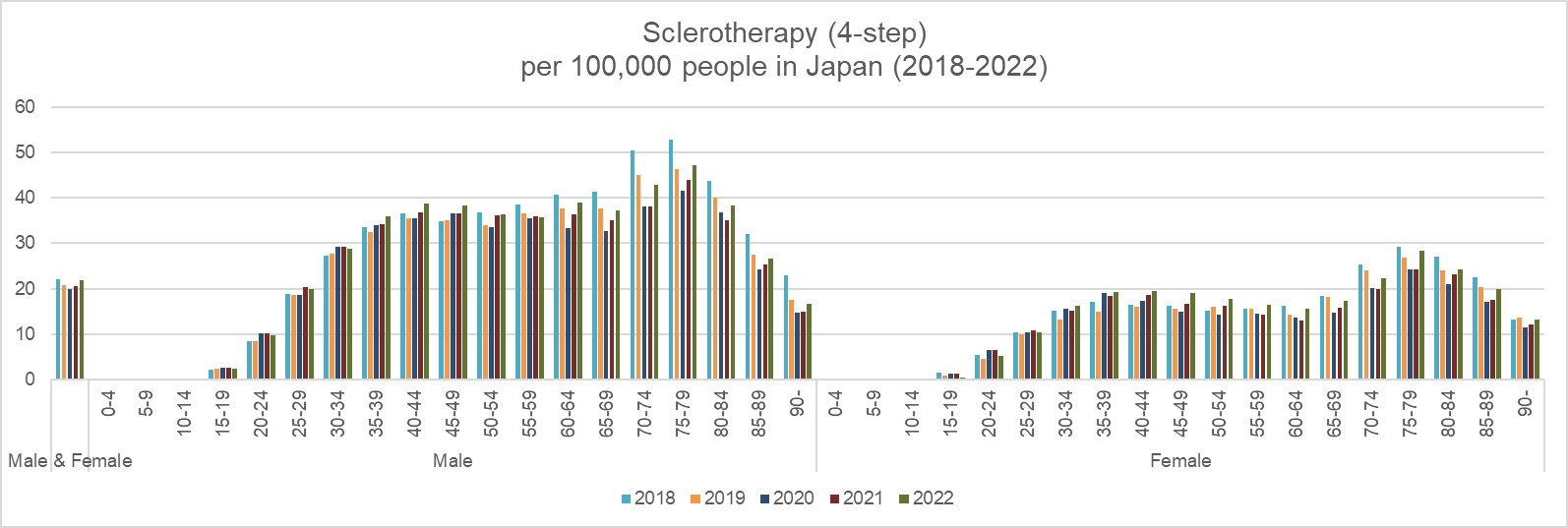


Supplementary Figure 6. Annual age-stratified number rate of invasive treatments for hemorrhoids (sclerotherapy (four-step)) per 100,000 people from 2018 to 2022 in Japan.


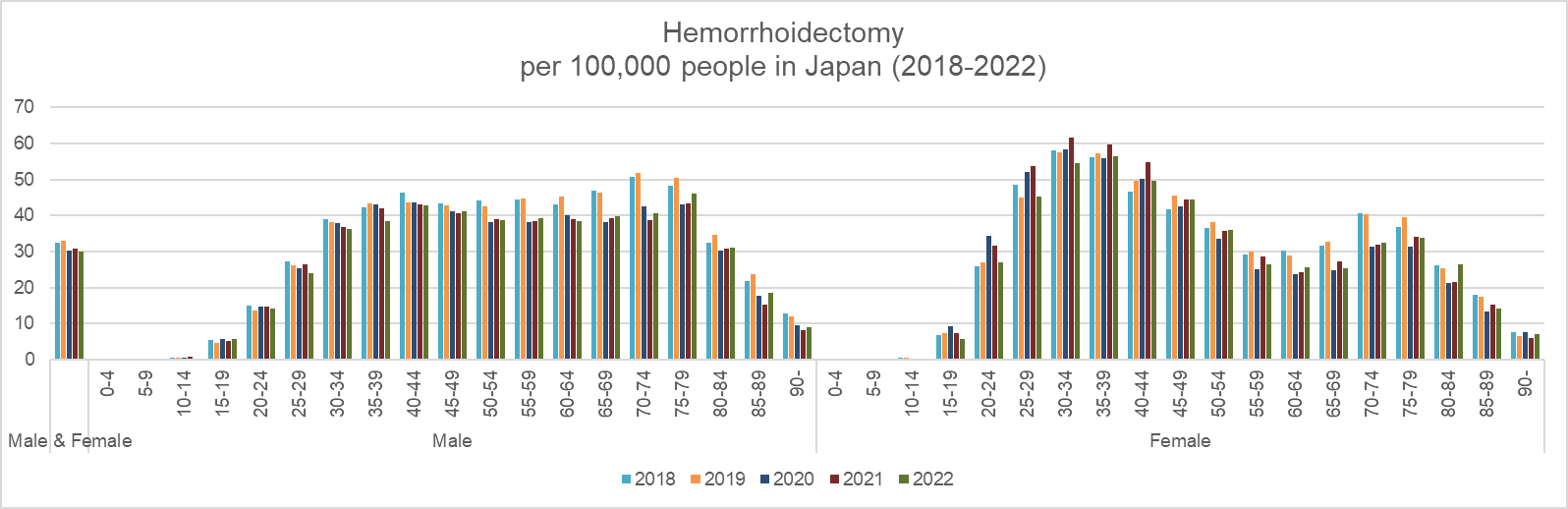


Supplementary Figure 7. Annual age-stratified number rate of invasive treatments for hemorrhoids (hemorrhoidectomy without sclerotherapy) per 100,000 people from 2018 to 2022 in Japan.


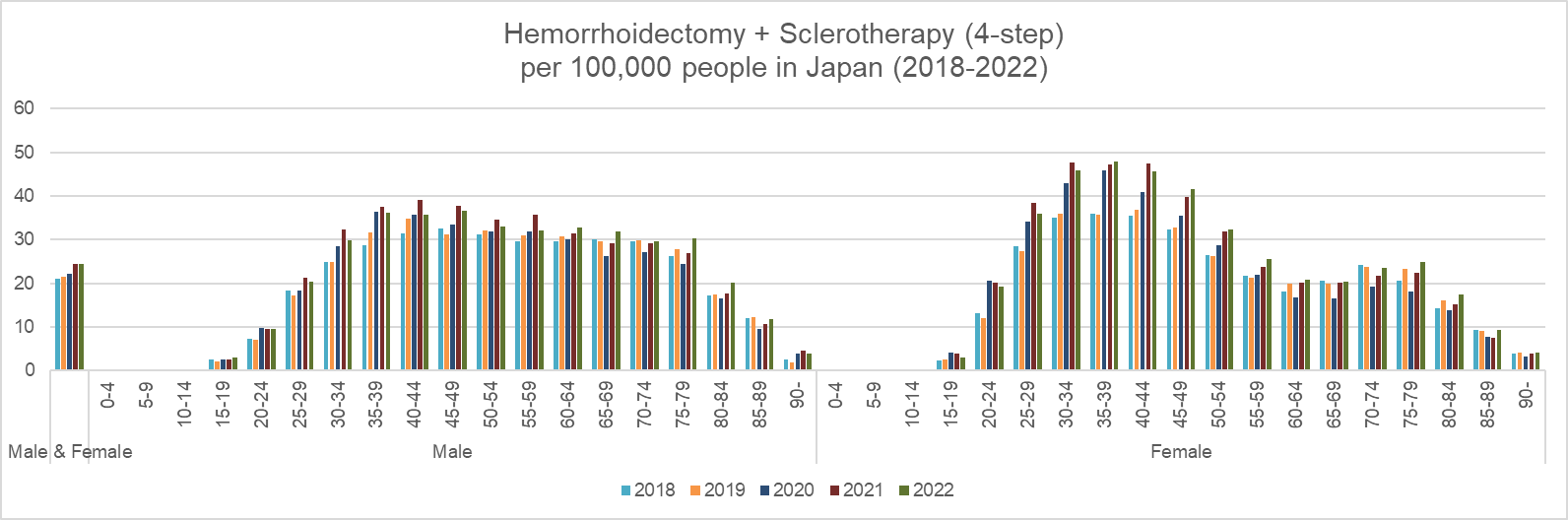


Supplementary Figure 8. Annual age-stratified number rate of invasive treatments for hemorrhoids (hemorrhoidectomy with sclerotherapy (four-step)) per 100,000 people from 2018 to 2022 in Japan.


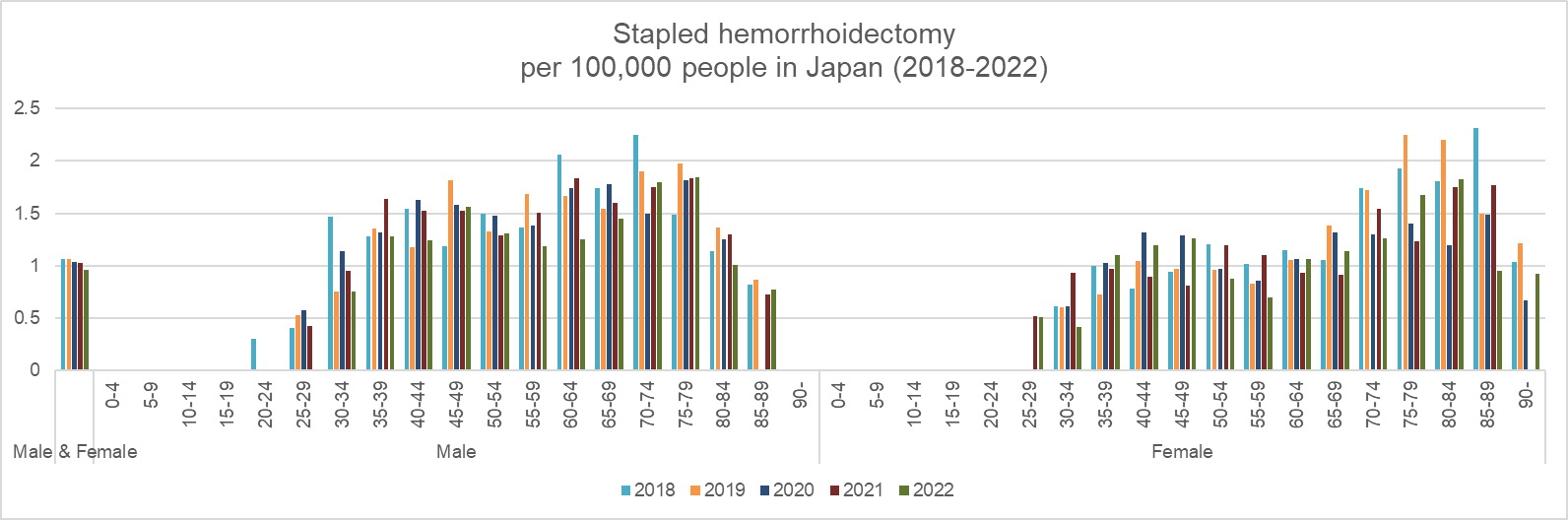


Supplementary Figure 9. Annual age-stratified number rate of invasive treatments for hemorrhoids (stapled hemorrhoidopexy) per 100,000 people from 2018 to 2022 in Japan.

Supplementary Figure 10. Birthrate per 1,000 female population stratified by mother's age.
